# Gaze-Based Intention Estimation for Shared Autonomy in Pick-and-Place Tasks

**DOI:** 10.3389/fnbot.2021.647930

**Published:** 2021-04-16

**Authors:** Stefan Fuchs, Anna Belardinelli

**Affiliations:** Honda Research Institute Europe, Offenbach, Germany

**Keywords:** intention recognition, shared autonomy, eye tracking, teleoperation, eye-hand coordination, Hidden Markov Models, human-robot interaction

## Abstract

Shared autonomy aims at combining robotic and human control in the execution of remote, teleoperated tasks. This cooperative interaction cannot be brought about without the robot first recognizing the current human intention in a fast and reliable way so that a suitable assisting plan can be quickly instantiated and executed. Eye movements have long been known to be highly predictive of the cognitive agenda unfolding during manual tasks and constitute, hence, the earliest and most reliable behavioral cues for intention estimation. In this study, we present an experiment aimed at analyzing human behavior in simple teleoperated pick-and-place tasks in a simulated scenario and at devising a suitable model for early estimation of the current proximal intention. We show that scan paths are, as expected, heavily shaped by the current intention and that two types of Gaussian Hidden Markov Models, one more scene-specific and one more action-specific, achieve a very good prediction performance, while also generalizing to new users and spatial arrangements. We finally discuss how behavioral and model results suggest that eye movements reflect to some extent the invariance and generality of higher-level planning across object configurations, which can be leveraged by cooperative robotic systems.

## 1. Introduction

Shared autonomy has recently emerged as an ideal trade-off between full autonomy and complete teleoperation in the execution of remote tasks. The benefits of this approach rely on assigning to each party the aspects of the task for which they are better suited. The lower kinematic aspects of action execution are usually left to the robot while higher-level cognitive skills, like task planning and handling unexpected events, are typically concurrently exercised by the human, in a blend that can entail different degrees of autonomy for the robotic part (Goodrich et al., 2013; Beer et al., 2014; Schilling et al., 2016). Considering the often large asymmetry in terms of degrees of freedom or kinematic capabilities between the user input controller (e.g., joysticks) and the robotic effector, shared autonomy eases the operator cognitive load and speeds up execution improving motion fluency and precision. Since the user is setting the goals and the ways to achieve them, this collaborative effort relies on the robotic partner to first recognize the current human intention (*intent recognition*) and only afterwards to decide how much to assist with the execution (*arbitration*). Intention recognition should thus happen as early and as naturally as possible for the user to be relieved of explicitly directing the robot and for the robot to timely initiate the assisting action. To this end, although several approaches have been proposed that rely on intent recognition from the user control input driving the robotic movement (Yu et al., 2005; Aarno and Kragic, 2008; Hauser, 2013; Javdani et al., 2015; Tanwani and Calinon, 2017; Yang et al., 2017), the most natural and timely way to predict intention both in assistive technologies and remote manipulation is certainly to use gaze cues, as reviewed in the next section. In light of the need to cope with sensorimotor delays (Miall and Reckess, 2002), gaze control itself in task-based scenarios can be considered as inherently predictive of a number of action-relevant aspects. Indeed, in moving our eyes we make use of knowledge- and sensorimotor-based experience (Belardinelli et al., 2016; Hayhoe, 2017; Henderson, 2017; Fiehler et al., 2019) to quickly retrieve the information needed to plan limb motion.

In this study, we focus on gaze-based intention prediction in teleoperating a robotic gripper in a simulated scenario, to investigate human eye-hand coordination under these conditions and to devise an intention estimation model to be later transferred to a real-world shared autonomy scenario. As a first setup for object manipulation, we concentrate on basic pick-and-place tasks as common in this kind of architectures (Javdani et al., 2015; Li et al., 2017; Jain and Argall, 2018, 2019; Shafti et al., 2019). Presented contributions are a behavioral assessment of eye-hand coordination in such scenarios and the design of two Gaussian Hidden Markov Model schemes trained on collected data, showing good generalizability across users and task configurations.

In the next sections, related work on gaze-based intention recognition is first reviewed; the experimental methods used in our setup and the devised models are further presented, followed by results obtained from behavioral analysis and model testing. We conclude by discussing emerged implications and future perspectives.

## 2. Related Work

That the task shapes the way we look at the world has long been known, as shown by Yarbus (1967). In that study, it was shown that depending on the question the viewer was trying to answer different scanning patterns were produced on the very same image. A number of studies have replicated and confirmed Yarbus' experiment and managed to invert the process and estimate the task from eye data above chance level (e.g., Borji and Itti, 2014; Haji-Abolhassani and Clark, 2014; Kanan et al., 2014). The most popular and effective techniques to compute the probability of a given task given eye movements and possibly their sequence entail Naive Bayes classifier, Hidden Markov Models, SVM, multivariate pattern analysis, and random forests (see Boisvert and Bruce, 2016, for a more complete review). The largely increased diffusion of wearable cameras and eye-trackers in recent years has triggered research on daily activities recognition as observed from an egocentric perspective (Yi and Ballard, 2009; Fathi et al., 2012; Ogaki et al., 2012), hence relying on eye, hand, head, and possibly body coordination (see Nguyen et al., 2016, for a full review).

Yet the approaches above are concerned either with passive information-seeking or with general activity recognition rather than with simple action or proximal intention recognition. Indeed, two basic types of intention have been postulated (Bratman, 1987): a mental state concerning intention for the future (*distal intention*), not necessarily situated in a specific spatial and temporal context, and intentionality for an immediate action (*proximal intention*). From a temporal perspective, a proximal intention is very close to the executed action. Thus, the boundary between proximal intention recognition and action recognition is at times rather blurry. The later an intention is recognized the more advanced the execution of the corresponding action might be.

In a recent study considering object aligning tasks in Virtual Reality (Keshava et al., 2020), it was shown how already simple features, such as the proportion of Points-Of-Regard (POR) on distinct Areas-of-Interest (AoIs) within the objects could constitute a sufficient oculomotor signature to discriminate between four different tasks, which could be classified well above chance. In human-robot collaboration often the robot partner is aware of the activity context and for effective cooperation, it just needs to detect the current action intention of the human partner to help them with it. Huang and Mutlu (2016) have proposed a method for anticipatory control which allows a robot to predict the intent of the human user and plan ahead of the explicit command. In the task considered, a robotic arm prepares a smoothie by picking the ingredients selected vocally by a human user looking at an illustrated list. By means of eye tracking the robot infers the user intention before they utter it and anticipates picking the intended ingredient: an SVM was fed a feature vector of gaze features for each ingredient, such as the number of glances, duration of the first glance, total duration and whether it was the most recently glanced item as predictors of the currently intended ingredient. Although such an approach seems simple and effective in this case the human user was carrying out no parallel visuomotor control task that could yield spurious fixations.

Within shared autonomy approaches, as a first attempt at integrating gaze input from the user, Admoni and Srinivasa (2016) put forward a proposal relying on Javdani's framework (Javdani et al., 2015), where the probability distribution over the goals (hidden states) is updated by considering both user's eye movements and joystick commands as observations in a Partially Observable Markov Decision Process (POMDP), using hindsight optimization to solve it in real-time.

In a further study (Aronson et al., 2018), the authors present an eye tracking experiment aimed at comparing user behavior within-subjects in different teleoperation modalities, namely with more or less autonomy. In the scenario of an assistive robot arm spearing food bits from a plate to feed an impaired user, by looking at partly manually annotated gaze behavior, two patterns of fixations emerged: monitoring glances, meant to check the translational behavior of the arm approaching the intended food morsel, and planning glances, which select the target morsel before starting the arm actuation, as in natural eye-hand coordination (Johansson et al., 2001; Hayhoe et al., 2003). Haji Fathaliyan et al. (2018) proposed a method to localize gaze on 3D objects by projecting the gaze vector on point cloud representations of the objects manipulated by a person preparing a powdered drink. Using Dynamic Time Warping barycentric averaging, sequences of gazed objects were obtained encapsulating the typical temporal patterns of object interaction that could be used for action recognition. Very recently, the same group used features extracted by this method to recognize action primitives in different activities (Wang et al., 2020). However, data were collected using natural eye-hand coordination, with participants executing the task themselves, which represents a different situation from a teleoperation scenario both on a perceptual and action control level. In the context of assistive robotics, a number of other studies have also considered gaze information (at times combined with multimodal interfaces, such as BCI and haptic feedback) to operate robotic limbs and wheelchairs (Schettino and Demiris, 2019; Zeng et al., 2020). Often in these cases, the gaze is used to implicitly but actively point the system to the object the impaired user wants the robot to interact with (Li and Zhang, 2017; Wang et al., 2018; Shafti et al., 2019).

Our study follows similar motivations as Aronson et al. (2018) and Wang et al. (2020) and complements those results, while not being aimed specifically at assistive applications, but rather trying to leverage human dexterity and eye-hand coordination to improve performance in teleoperated manipulation tasks. To investigate human oculomotor behavior during teleoperation in a more controlled scenario and with a more natural input interface, we designed an experiment in simulation, where the participant would control the remote robot arm by means of their own arm movements via motion tracking. We reasoned that this would produce more natural scanpaths and reaching behavior, without the cognitive overload of a controller with few DOFs, but still showing how the user copes on a sensorimotor level with the task of controlling a remote arm. These behavioral cues were further collected to train a proof-of-concept model able to predict the current intention in pick-and-place tasks in similar teleoperated scenarios, to be later deployed in a real-world setup^1^. Since many teleoperation scenarios relay visual input through a camera, we displayed the scene on a screen and used eye tracking glasses to retrieve the (POR) on the 2D display.

In our approach, we plan to work with multiple objects and to recognize different sequential sub-tasks, hence we chose to model scanpaths via Hidden Markov Models (HMM), which present the benefit of considering the temporal dimension of the gaze shifts and can better deal with spurious fixations and gaze samples and varying eye tracking frequency (Belardinelli et al., 2007; Coutrot et al., 2018; Boccignone, 2019). Our experimental setting and the intention estimation model are detailed in the next sections.

## 3. Behavioral Experiment Methods and Analysis

### 3.1. Participants

This study has been conducted after the outbreak of the COVID-19 pandemic. Hence, a number of participants suitable for this kind of study could not be recruited and to minimize infection risks only associates of the Honda Research Institute participating in this project were asked to take part in data collection on a voluntary basis (*N* = 4, including the authors). We complied with the measures of the *Occupational Safety and Health Standard* emanated by the German Federal Ministry of Labour and Social Affairs by keeping a safe distance and wearing face masks. The study was approved by the Bioethics Committee of Honda.

Participants had normal or corrected-to-normal vision, were all right-handed, and gave informed consent to participate in the study.

### 3.2. Experimental Setup and Procedure

**Figure 1:**
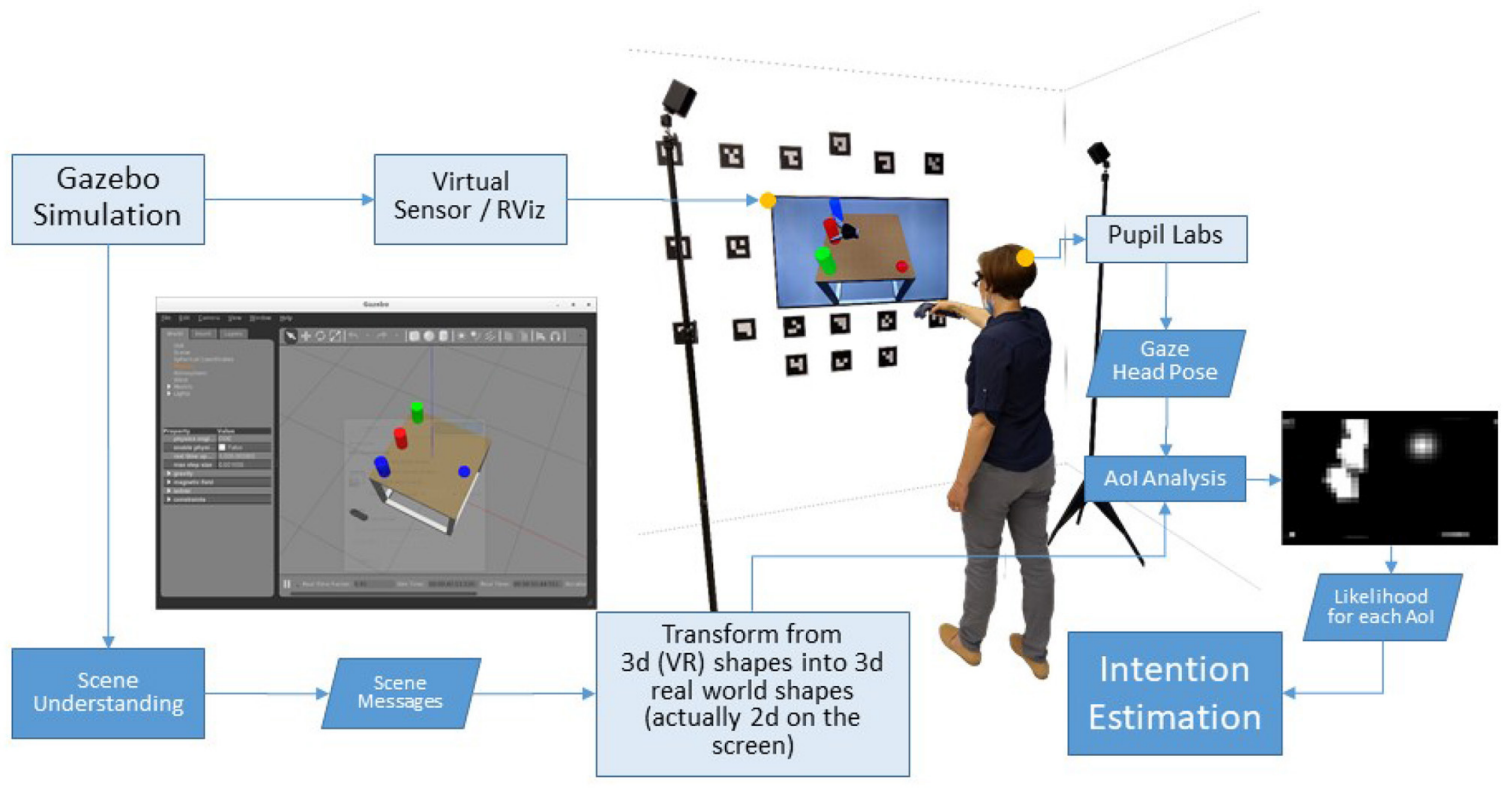
The experiment was carried out in a simulated scenario. The Gazebo simulator features physics and a virtual sensor, with its measurements rendered on the screen. The user controls the Pick-and-Place-Task with a HTC Vive controller, tracked by the Vive Lighthouse system. At the same time, the Vive controller and a Pupil Labs eye-tracking system are used to estimate the human sensorimotor state.

The experiment was carried out in a simulated scenario created with the Gazebo Simulator^2^ (see Figure 1). The scene was captured with a virtual sensor and displayed on a widescreen (1.21 × 0.68 m) with HD resolution in front of the participant, who was standing at a distance of about 1.5 m. Participants wore a binocular Pupil Core eye-tracker by Pupil Labs, working at 100 Hz with a reported accuracy of 0.6°. They also held in the right hand the HTC Vive controller, tracked by the Vive Lighthouse system for input control in the teleoperation task. All physical devices and surfaces were sanitized after each use. After instructions, participants were required to wear the eye tracking glasses, to adjust the eye and scene cameras according to the experimenter's directions, and to perform a 5-point calibration.

**Figure 2:**
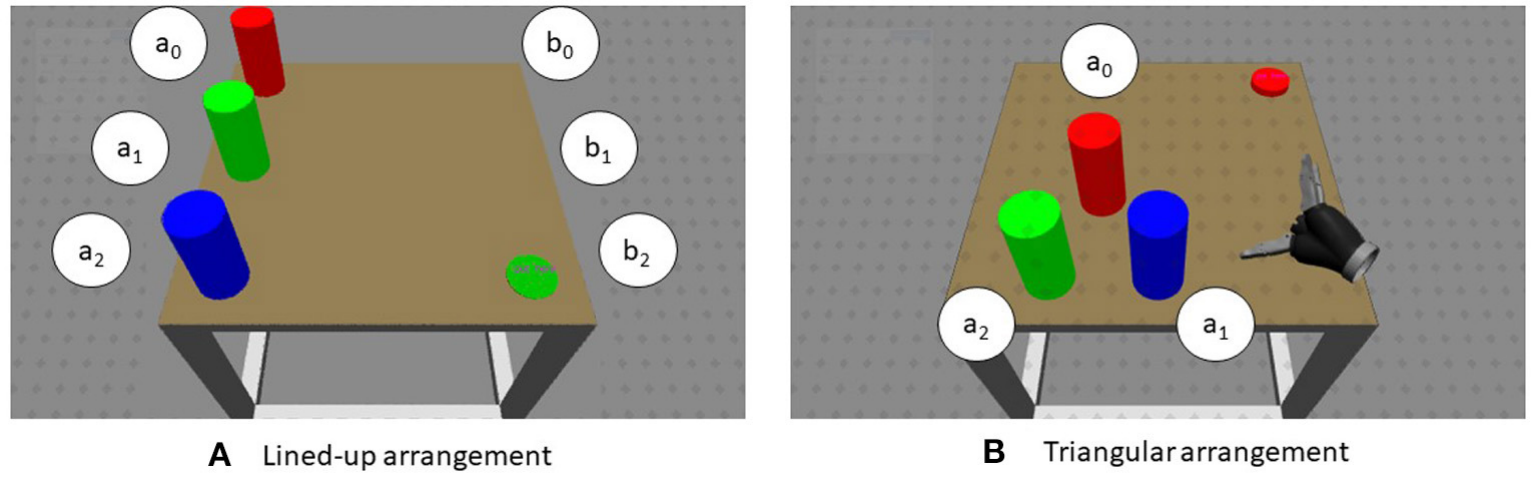
Example of scenes used in each trial. The objects to pick up were displayed lined up on the left side (or in a triangular arrangement) in three different colors while the disc on the right could similarly appear at each of three positions on the right side. The color of the disk signified which cylinder was to pick up, the position of the disk denoted the position for the placing down. The white disks are just shown here to label the picking and placing positions. **(A)** Lined-up arrangement. **(B)** Triangular arrangement.

The experimental stimuli consisted of three cylinders presented in two configurations (in different blocks): either aligned on the left side of a table (numbered as follows: 0 for the top, 1 for the middle, 2 for the bottom of the table) or at the vertices of a virtual triangle (0 for the top vertex, 1 for the bottom right, 2 for the bottom left; see Figure 2). Colors were permuted anew in each trial. Along with the cylinders, a disk would appear on the right side of the table, at one of three positions (denoted as: 0 top, 1 middle, 2 bottom). The disk specified the current pick-and-place task: the color indicated which cylinder to pick up and the position of the disk where the cylinder had to be placed down on the table. The task would be executed by a robotic gripper in the virtual scene, operated by the participant's movements. The position and orientation of the Vive controller grasped by a user's hand were tracked and mapped onto the gripper. Just the robot hand was visible and could be controlled by the participant as the own hand. No robotic arm kinematics was simulated in the mapping of the movement. Participants were required to reach with the controller in their hand toward the target and to grab it by pressing the button on the controller under the index finger. They had then to move the cylinder to the other side and release it on the place position, in so ending the trial. Between trials, a resting time of 5 s was given, followed by a fixation cross and indications on how to move the controller back to the rest position. As soon as the controller reached the starting position, the next trial started.

The cylinders were 20 cm high and with a radius of 5 cm. In the lined-up configuration, they were placed 30 cm apart, while in the triangular configuration cylinder 1 and 2 were about 21 cm apart and both were 22.6 cm apart from the cylinder in position 0. The robotic gripper was about 16 cm long from the wrist to the midpoint between the fingers.

### 3.3. Design and Data Processing

We designed two different arrangements of the cylinders since we hypothesized that the positions of the objects would require different movement trajectories and oculomotor strategies. In this way, we could investigate the impact of the spatial arrangement of the items on the gaze behavior.

Each trial consisted of a reach-and-grasp phase and a transport-to-place phase to the placing target position. The two phases are separated by the gripper grasping the picking target. In this sense, in the following, picking times are considered as the time from the start of the trial to the grasp event detected via button press. The transport phase spans the time from the grasp event to the end of the trial, i.e., when the gripper button was released and the cylinder in hand was within 10 cm of the placing disk. The tasks are defined by the positions of the respective targets, e.g., *pick_0* for picking at the pick position 0 or *place_1* for placing at the place position 1. In each trial the target pick and place positions were randomly generated. This has led to an uneven number of pick-place target combinations for each participant. Lined-up and triangle arrangements were probed in separate blocks. Specifically, the final dataset consisted of sequences containing for the lined-up configuration 63 examples of *pick_0* and *pick_1*, 54 of *pick_2*, 60 of *place_0*, 55 of *place_1* and 65 of *place_2*. For the triangular configuration the dataset contained 35 examples of *pick_0* and *pick_1*, 27 of *pick_2*, 26 of *place_0*, 30 of *place_1*, and 41 of *place_2*.

Instead of working with relative eye coordinates, we used the fiducial markers and the scene camera of the Pupil Labs device to localize the eye-tracking-glasses in the scene w.r.t. the world and screen, respectively. Fixations represent a very popular cue in eye-movement data analysis and might seem an obvious choice in this intention estimation application. The parameterization of a fixation identification method, however, might be very arbitrary. Usually, thresholds are chosen to determine when exactly fixations start and when they end. Thus, the parameters of a fixation identification algorithm can have a dramatic impact on our higher-level analyses (Salvucci and Goldberg, 2000). Further, the system will be required to work online eventually and online fixation recognition is not always accurate while further increasing the computational load. The temporal information related to dwelling time in the AoIs (the objects of interest in the scene) during fixations is still learned and considered by the HMM all along.

**Figure 3:**
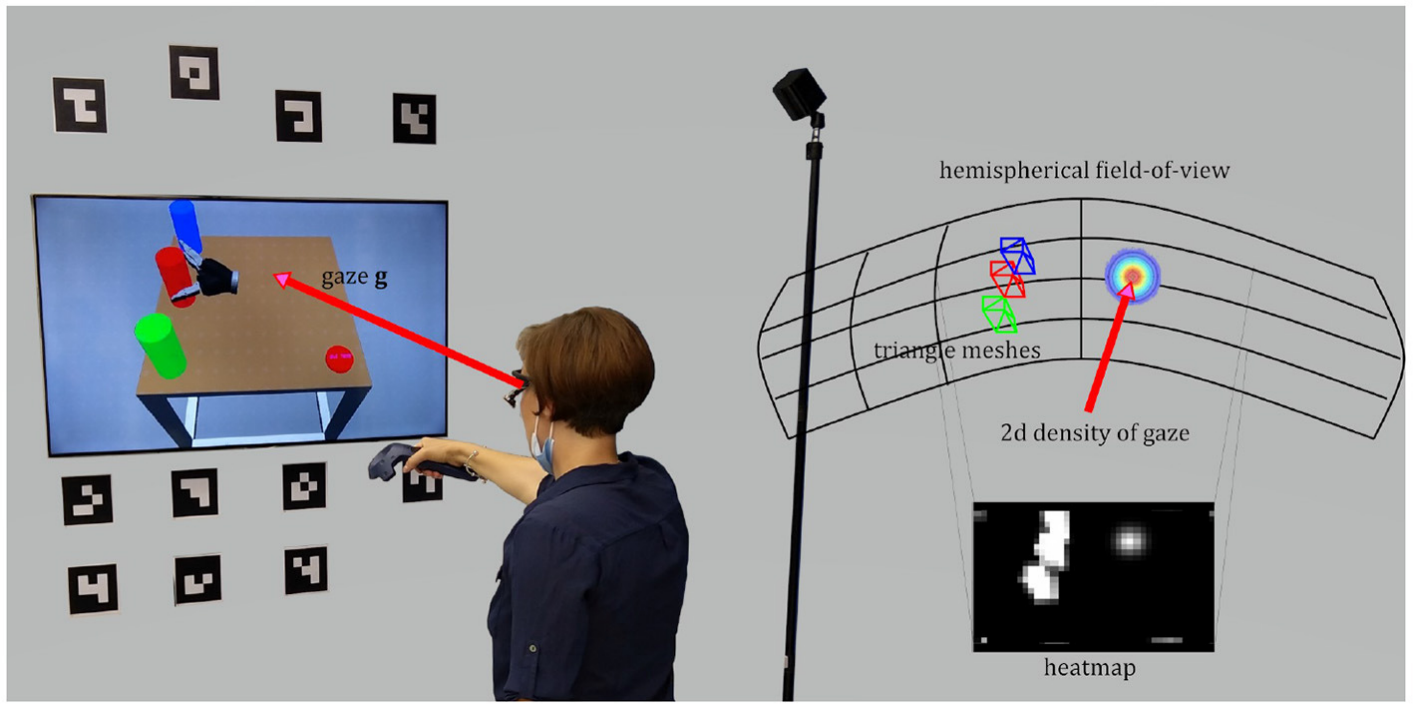
The user's field-of-view is approximated by a hemispherical heatmap. The density of a 2d normal distribution centered on the point-of-regard represents the gaze and its uncertainty. The surface integral over the triangles of a certain object is the likelihood of this AoI.

For these reasons, we decided to work with gaze samples that were mapped on the scene according to the following approach (depicted in Figure 3). A heatmap with a discrete resolution represents the hemispherical field-of-view of the participant. In this case a sampling of 1° is used and the heatmap comes with a resolution of 180 by 90 px. The user's eye gaze **g** is represented by a two-dimensional normal distribution and the density is plotted onto the heatmap with gaze uncertainty σ and location centered on μ^3^. The choice of the size of σ might depend on the accuracy and precision of the eye tracking measurements. Here, we set σ = 2 ° which is in accordance with the size of the human fovea. All potential scene objects are represented as triangle meshes with a bounding box made of at least 12 triangles. The pose of the objects is delivered by a scene understanding module and given the localization of the eye tracking glasses, the object poses can be transformed into the head coordinate system. Mesh triangles, that are visible to the user (i.e., normal of triangle directed toward the user), are plotted onto the heatmap. The surface integral of the density function over these triangles represents the likelihood that this area is regarded by the user. The complete likelihood (of each object to be regarded by the user) is the sum of all visible triangles the object is made of. In order to not overemphasize large objects, all likelihoods are normalized by their visible areas. For each object an Area-of-Interest was defined, for a total of seven AoIs: for the picking objects the areas {*a*_0_, *a*_1_, *a*_2_}, for the placing positions the areas {*b*_0_, *b*_1_, *b*_2_}, plus an area *R* for the robotic gripper.

As a result, this so-called *Area-of-Interest-analysis* provides for every gaze sample **g** a feature vector **F** entailing the likelihood computed for each of these AoIs:

(1) $$F_{t}=\{\text{P}(\text{AoI}=a_{0}|g_{t}),\text{P}(\text{AoI}=a_{1}|g_{t}),...,\text{P}(\text{AoI}=R|g_{t})\}\;.$$

These were logged along with the current hand position and robot gripper position and with the current grasping state (defined as the binary state of the grasping button). Trial samples were further labeled with a Boolean feature to state if the trial was successful. Indeed, if the grasp failed for any reason multiple grasp attempts could be observed or none at all if the cylinder was toppled down and fell off the table.

### 3.4. Behavioral Analysis

To get a better picture of the gaze behavior during the presented task, we looked at some behavioral measures, seeking confirmation of some of the patterns described in Aronson et al. (2018). Due to the low number of participants thus far, we could not perform an inferential statistics analysis within subjects to test any hypothesis, hence for the most part we report a descriptive analysis computed over the whole dataset depending on the different tasks.

**Figure 4:**
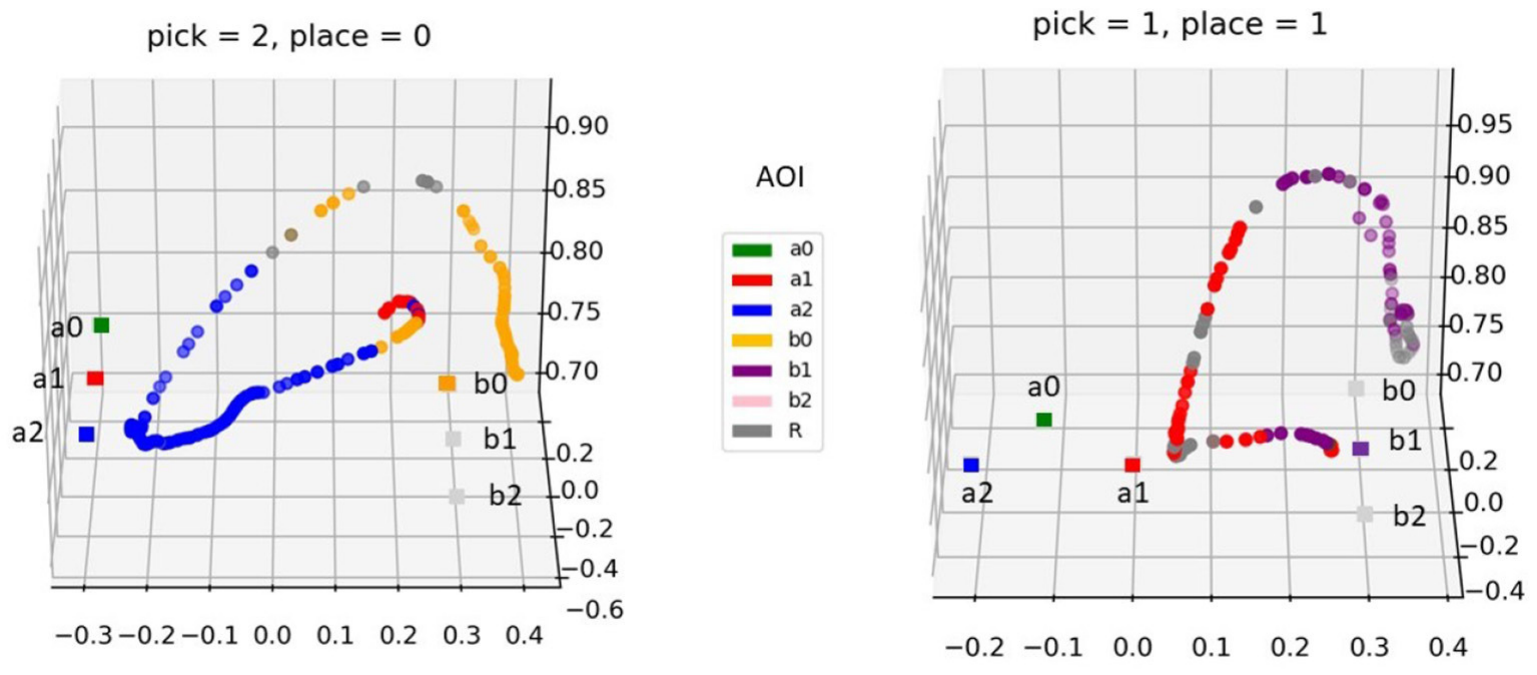
Two exemplary trajectories of the hand during the pick-and-place tasks (left: pick in position 2 and place in position 0; right: pick in position 1 and place in position 1). The movement samples are colored with the currently gazed AoI (see legend). The square markers denote the picking and placing positions: the former are here denominated and colored as the respective AoIs, for the latter only the current placing target is presented in color (the other targets are in light gray since they were not visible but their position is shown for reference).

Two exemplary trajectories for different pick-and-place tasks and object configurations are depicted in Figure 4. At any time, the AoI collecting the highest likelihood is considered the one currently looked at. Upon motion onset the AoI corresponding to the place target (whose color determines also the picking target) is glanced. This is a planning glance, as defined in Aronson et al. (2018). Right afterward the gaze moves to the picking target. It must be noted that this glance at the place location is due to the way the task is designed. Possibly, it would not be observed if the picking target was communicated to the user in another way, e.g., verbally, with the placing target displayed as a gray disk, for instance. During the transport phase, the gaze targets the placing target. During both the reach-to-grasp phase and the transport-to-place phase the robot AoI (gray) is checked in a monitoring pattern, to make sure the gripper is moving in the intended direction.

**Figure 5:**
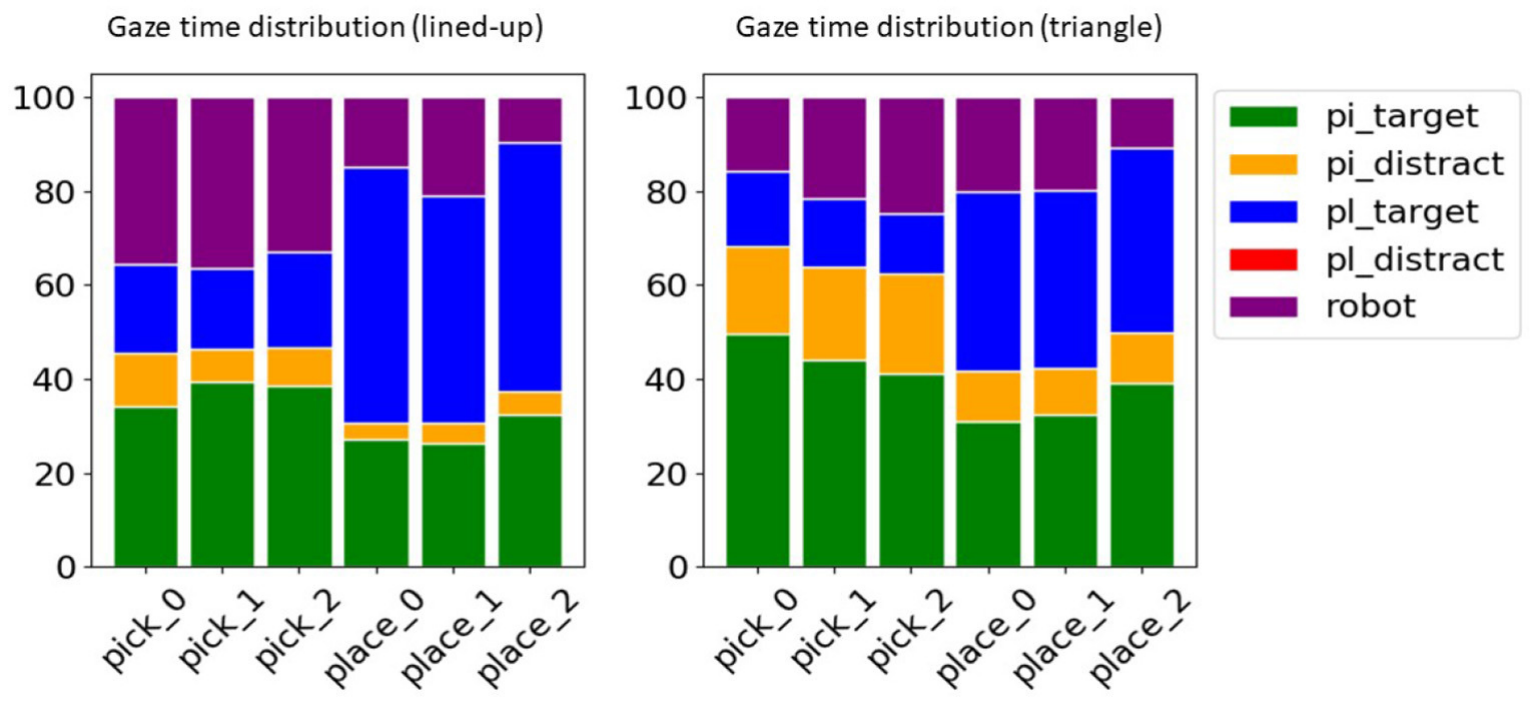
Relative distribution of the time the gaze spent on semantic AoIs across tasks for the lined-up (left) and the triangle arrangement (right). In the pick tasks the respective picking AoIs (pi_target) are more looked at, in the place tasks the respective placing AoIs (pl_target). The other AOIs were summarized in the pick distractors (pi_distract, i.e., the cylinders not to be picked up), place distractors (pl_distract, i.e., possible place-down locations other than the target), and the robot hand (robot).

For each trial, we consider two intentions/(sub)tasks, one picking and one placing intention, separated by the keypress triggering the grasping. To get a more complete overview of the time the gaze spent in different AoIs across tasks, the relative time distribution of gaze on each AoI was computed and is presented in Figure 5. To make the picture easier to interpret, we considered that for each intention there are actually just five semantic entities that are relevant to describe the gaze behavior, namely: the pick target (e.g., *a*_0_ for *pick_0*), the pick distractors (e.g., *a*_1_, *a*_2_ for *pick_0*), the place target (e.g., any of the *b*_*i*_ AoIs depending on the current task), the place distractors (e.g., any of the *b*_*i*_ AoIs that are not the target) and the robot hand. Analogously for the place intention, the place target would be the specific AoI related to that task, while the pick target could be any of the picking positions and the distractors are the pick and place AoIs that are not the current pick nor place target of the trial.

**Figure 6:**
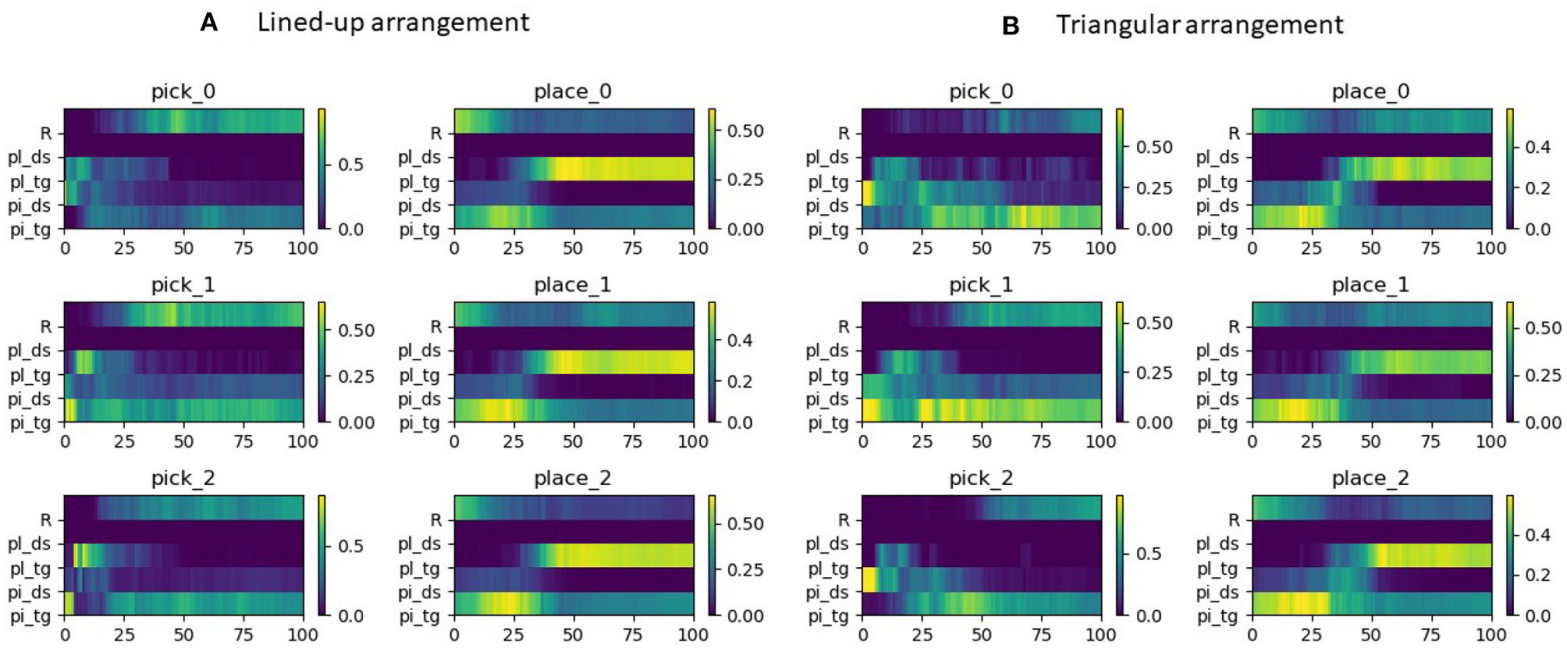
Gaze distribution on a normalized time axis across semantic AoIs. Time = 0 signifies starting of the trial and time = 100 the closing of the gripper on the object for the pick sequences. This last event represents the start of the normalized time axis for the place sequences, with 100 indicating the end of the trial. **(A)** Lined-up arrangement. **(B)** Triangular arrangement.

As can be noted from Figure 5, for either task of pick or place, the distributions of the gaze time share a common pattern on a semantic level, i.e., the target of the task is longer dwelled on. Thus, these tasks can be distinguished as each task corresponds to a different semantic target (picking or placing objects). Yet the distributions are also distinctive within each task, considering that each action target is the AoIs related to the task target, i.e., the corresponding *a*_*i*_ position in the pick tasks and the corresponding *b*_*i*_ position in the place tasks. In the pick tasks the place target is briefly looked at to learn the pick target, while in the place tasks the pick target receives also some attention since, after pressing the button for the grasp and in absence of any haptic feedback, the gaze checks that the object is correctly grasped. This evolution in time can indeed be appreciated better in Figure 6, where trials across intentions were averaged on a normalized time axis between the start of the trial and the grasp for the pick trials and between the grasp event and the end of the trial for the place trials. In these latter, independently of the place target, it can be noted how for about the initial 30% of the placing task the pick target is still looked at, to visually check whether the object is lifted up with the gripper, hence confirming the grasp was successful. Interestingly, in both configurations and tasks also the robot effector receives a discrete amount of gaze time and in the pick trials shares a lot of gaze distribution with the pick target. Of course toward the end of the pick and place trials the gripper is close to the pick/place targets and the gaze can have both within the fovea or in the parafoveal space and monitor them at the same time. Still, in natural eye-hand coordination, the hand instead is rarely looked at (Johansson et al., 2001) because proprioceptive information and peripheral vision usually suffice to monitor it. This suggests that in this teleoperation scenario the unusual sensorimotor mapping from the arm and controller to the three-fingered robotic gripper, especially considering the grasp pose, and possibly some delay in the tracking makes the user uncertain about the effector movements and current pose. Participants, thus, produced multiple monitoring glances (Aronson et al., 2018) during the movements to visually adjust the effector trajectory and pose. However, in general, the distributions looked rather distinctive across tasks, suggesting that it could be possible to reliably discriminate among them, while they looked rather similar across picking configurations, hinting to the possibility to generalize from one to the other. The pick distractors are looked at especially during picking, since the gaze checks the neighboring cylinders in order to decide the best grasp and in order not to collide with them. This is especially the case in picking at position 1 in the lined-up case and overall in the triangle configuration since the cylinders are all close to one another. The place distractors do not receive any attention since in each task only the target position was made visible with a disk in this experiment (see Figure 2).

To gain further insight into the difficulty of the task, we looked into the number of failed trials across picking tasks. Error rates were computed for the three pick tasks in the two configurations. The picking action in the lined-up configuration was successful in the 71.4% of *pick_0* cases, 88.9% for *pick_1* trials, and 79.6% for *pick_2*. In the triangular configuration, the grasp was successful in 68.6, 88.6, and 85.2% of cases, for the same picking cases, respectively. The users could accomplish the task in the vast majority of the cases, but a significant number of failed grasps occurred when picking at position 0 in both configurations.

This could be the case for different reasons. In the lined-up configuration, the 0 position is the rearmost and the one requiring to stretch the arm until the furthest edge of the table. However, 3D depth on a 2D plane is badly estimated, especially in a virtual scene where size cues are more difficult to gauge and the own body could not be used as reference either. In the triangle configuration, the 0 position is closer to the user, yet the other two objects are placed in front of it, requiring to pick the cylinder from above or—for a right-handed user—trying to avoid the cylinder in position 1 going around it. The depth estimation difficulty could yet be ameliorated in a virtual reality set-up.

**Figure 7:**
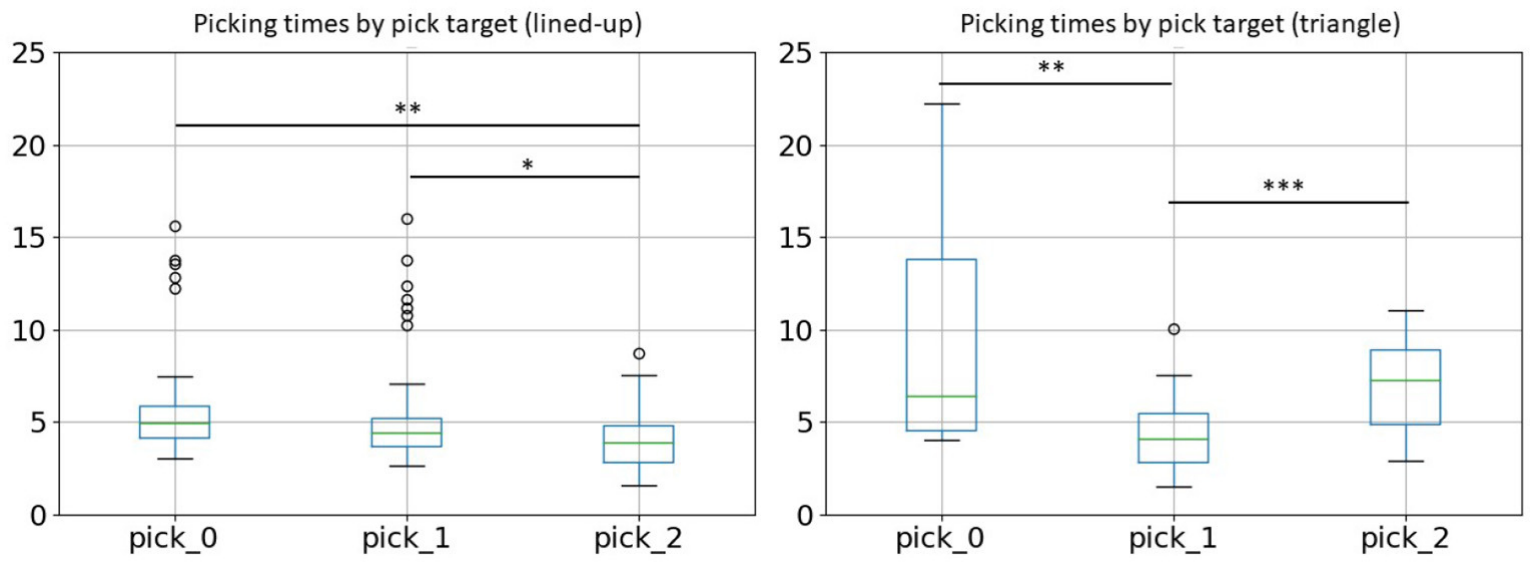
Picking times across picking position for the two object configurations. The rearmost position requires a longer reaching time in both configurations, also due to difficult depth estimation. In the triangle configuration the forefront position on the left (pick_2), besides the rearmost position (pick_0), requires a longer picking time.

A similar pattern emerges also looking at picking times. In this case, we considered only successful trials since in a failed trial no grasp or more than one grasp could occur. Looking at Figure 7, it can be noted again that the rearmost position requires the longest reaching time. The difference is significant between position 0 and position 2 [Bonferroni corrected Welch's *t*-test, *t*(67.11) = 3.56, *p* = 0.002] and between position 1 and 2 [*t*(88.34) = 2.94, *p* = 0.012]. In the case of the triangular configuration, also items in position 2 require a more careful movement, since a right-handed person needs to mind avoiding the cylinder in position 1 when approaching the cylinder in position 2 with the open gripper [position 0–1: *t*(27.24) = 4.02, *p* = 0.001, position 1-2: *t*(40.54) = −4.15, *p* < 0.001].

## 4. Computational Modeling and Results

### 4.1. Modeling Intentions With Gaussian HMMs

**Figure 8:**
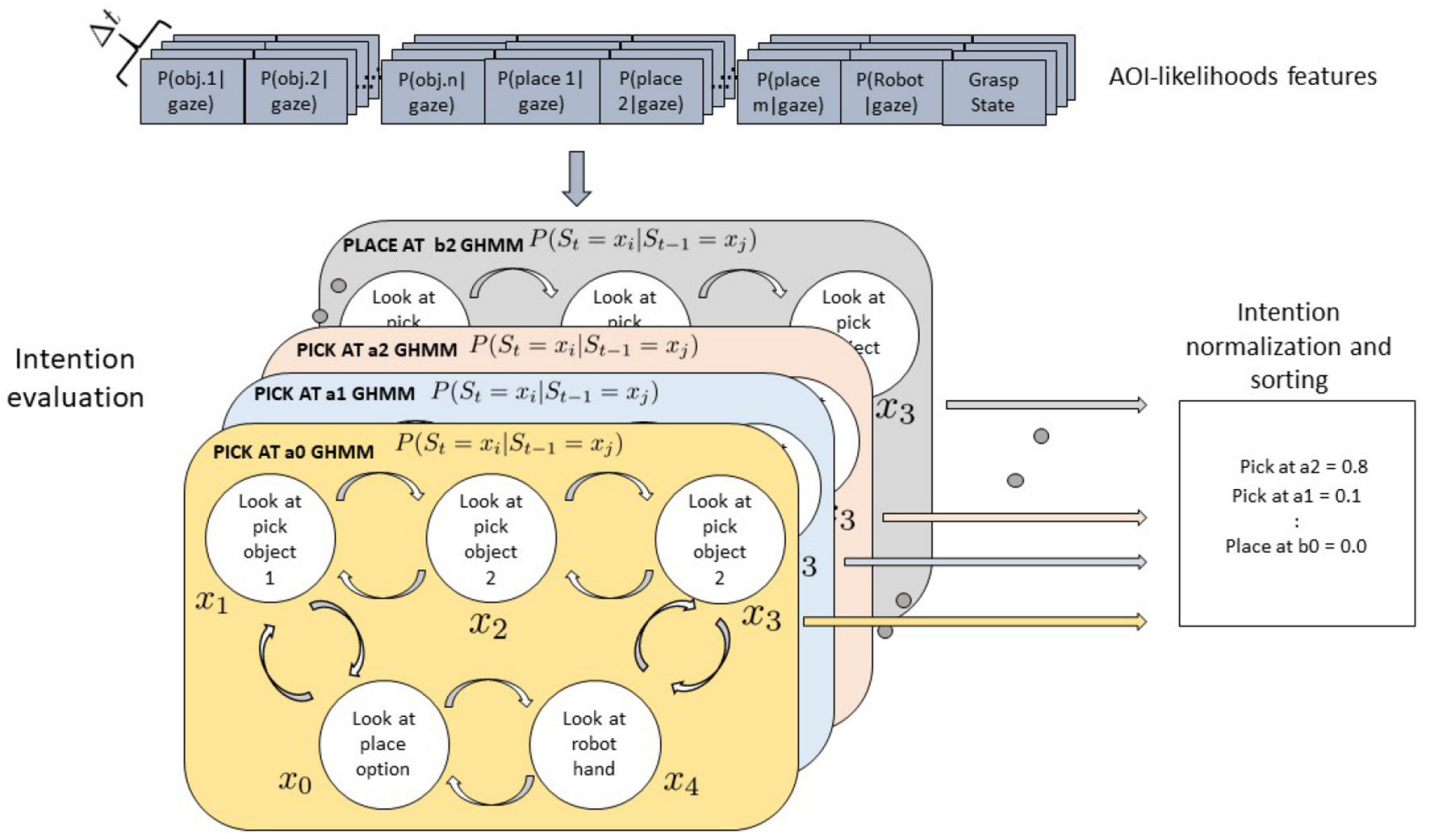
Gaussian Hidden Markov Models for a Pick-and-Place-Task. Five hidden states **X** are shown which might represent the perceptual state of the user looking at the object to be picked, at the robot, at the placing position target, or at the teleoperated robotic hand. Arrows between states represent transition probabilities: for the sake of legibility here only transitions between adjacent states are shown but actually all states are fully connected. A model is defined for each pick-object and place-at-location intention. Each model receives as input a sequence of vectors of AoI likelihoods, representing the probability of the object under the gaze distribution at different time steps. The emissions probabilities defining the probability of each state to emit the observed features are learned from the data and assumed Gaussian. Each model outputs a likelihood of the corresponding intention after observing the current sequence of features.

Our approach aims at predicting the proximal intention, i.e., the current action and the involved object. Gaze not only comes with a specific pattern during action execution but also provides early cues that indicate parameters of a pick and place task, such as which object to pick or where to place it down. These parameters are defined by the proximal intention (Bratman, 1987; Pacherie, 2008). The temporal gaze pattern can be represented with a Gaussian Hidden Markov Model (see Figure 8). The hidden states **X**(*t*) describe the internal intention process and might relate to *looking at the target object* or *looking at the placing position*. However, this is just an assumption, while the hidden Markov process drives an observable gaze sequence **Y**(*t*). The gaze sequence is described by the sequence of AoI likelihoods as derived from the multivariate Gaussian distribution (see section 3). The distribution of these AoI likelihoods at a particular time is governed by the emission probabilities of the hidden Markov process given the state of the hidden variable at that time. This approach is independent of the gaze sequence length, i.e., observation sampling and execution velocity, as long as the sequences are scaled linearly.

We defined six intentions to be recognized: three pick-up intentions (for each of the three cylinders) and three place intentions (for each of the three placing positions). Hence, six HMMs have been configured with five internal states. The observation vector of an HMM comprises eight components: the AoI likelihoods of the three cylinders, the AoI likelihoods of the three possible placing positions, the AoI likelihood of the robot, and the trigger button state of the Vive controller. The transition and emission parameters were learned by each HMM, which was fed the respective training sequences (between 19 and 31 observations sequences for each model for a total of 160). These sequences were all performed by two users. The training was done offline with data only from the lined-up arrangement and only successful pick-and-place tasks (no multiple grasp attempts, no toppling or dropping of the cylinders).

**Figure 9:**
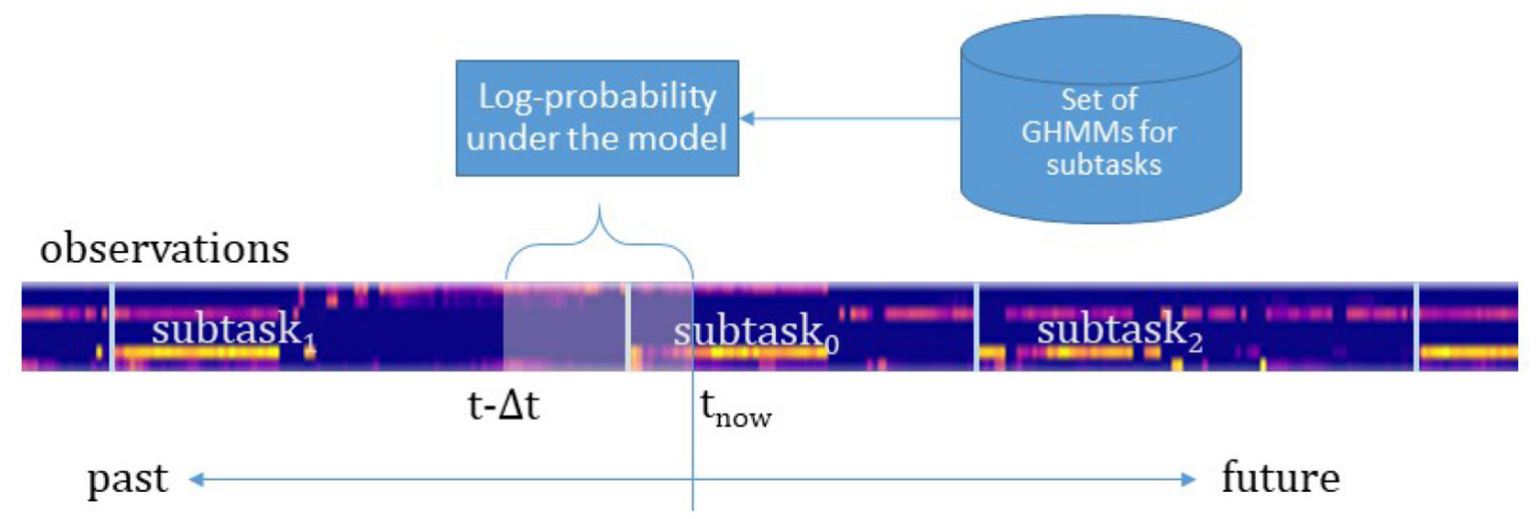
The observations made in the last Δ*t* s are used to compute the log-probability of these observations under each of the trained GHMMs. Here, an example sequence of subtasks with the observations is shown. The feature vectors are color-coded, vertically plotted, and concatenated (generating the bluish bar). The length Δ*t* of the time window decides on the accuracy and the earliness of the intention predictions.

Figure 9 sketches the online intention recognition approach. At every time step t the observations from the last Δ*t* s are used to compute the log-probability of these observations under each of the trained HMMs. The HMM with the highest log-probability exceeding a given threshold (κ = 0) is taken as prediction of the respective intention. If no model scores over the threshold, no intention is confidently recognized. The offline training and the online recognition are implemented in Python with the help of the *hmmlearn*-library^4^.

The performance of this approach is tested on data from four users (between 17 and 28 observation sequences for each intention, respectively, for a total of 128 sequences). The testing data comprised unseen sequences in the lined-up arrangement from the two users used for training plus sequences from two additional users. Moreover, testing was done also on sequences from blocks with triangular arrangement (between 19 and 33 sequences for each intention, for a total of 156).

### 4.2. Intention Recognition Results

**Figure 10:**
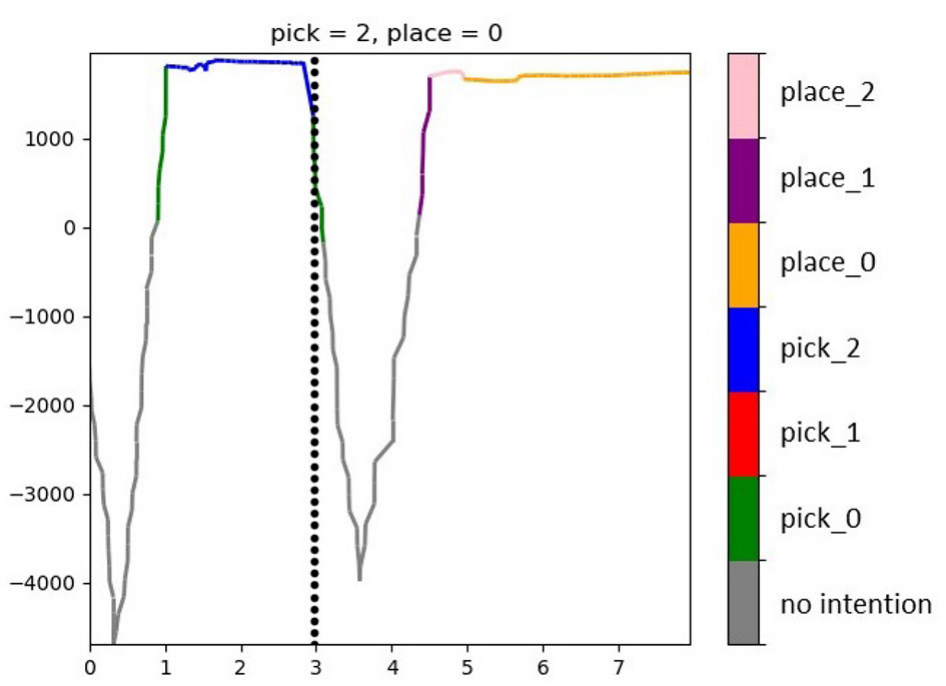
Likelihood of the predicted intention for a trial picking in position 2 and placing in position 0. The black dashed vertical line shows the time of the grasp event. At any time the likelihood of the currently highest model score is depicted in the color associated with the respective intention (see legend). If the highest score is below the threshold κ = 0, no intention is accepted (gray line).

To evaluate the intention recognition performance, we looked on the one hand at how accurate was the prediction whenever a prediction was indeed available (that is, the proportion of correct predictions over the overall number of delivered predictions). On the other hand, as stated in Ellis et al. (2013) and Wang et al. (2020), there is a trade-off between accuracy and observational latency. Indeed, the more evidence is accumulated before making a prediction, the more accurate the prediction is going to be. Yet, in the case of systems that should act on that prediction, the earlier this comes the better. To this end, maximizing accuracy can be at odds with minimizing latency. We looked also at this kind of latency and call it *predictability*, because of the way it is operationalized. Predictability refers to the fraction of action execution time where an intention is confidently recognized (regardless of whether right or wrong), defined as the ratio between the number of action samples for which an intention estimation is over the threshold κ and the overall number of samples in the action. At the beginning of a new trial when the gaze is still wandering between the placing target to check its color and the pick target, perhaps also checking the pick distractors, it is most likely that the models cannot deliver a confident enough prediction. Similarly, in the transport phase, after checking the successful grasp, the gaze quickly moves from the pick target to the place target. In this case, the observed time window might contain both samples related to the grasped object and to the place destination, hence even the highest-scoring model might deliver a very low likelihood score (under the threshold). This can be appreciated in the example in Figure 10: in the beginning of the trial no model reaches a confident enough likelihood score, but as soon as evidence is accumulated in favor of a picking action, the winning likelihood oversteps the threshold. At first, the wrong picking intention is predicted while later the correct model reaches the highest likelihood. A similar course is displayed after the grasp event, with the likelihood going down and then rising again in favor of a placing action.

**Figure 11:**
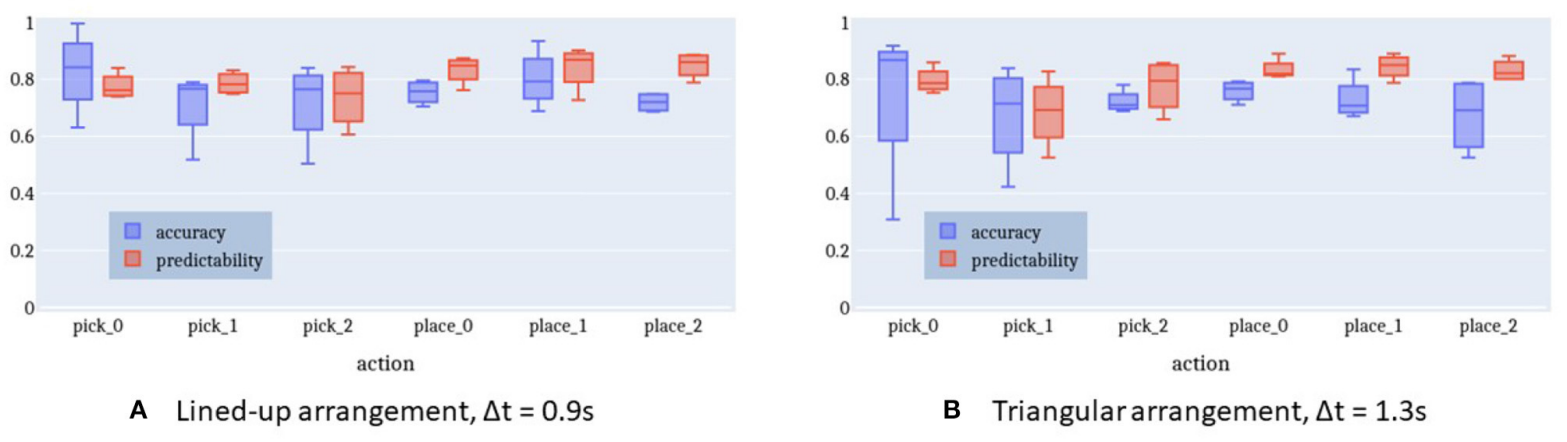
Accuracy and predictability of online intention recognition for lined-up arrangement and triangle shape arrangement. **(A)** Lined-up arrangement, Δt = 0.9 s. **(B)** Triangular arrangement, Δt = 1.3 s.

Figure 11A shows the accuracy and predictability of the intention recognition when using a time window of 0.9 s for the lined-up arrangement. On average, the HMM with the best log-probability being above the given threshold (κ = 0) indicates the correct intention in 78% of cases (chance level = 16.7%).

**Figure 12:**
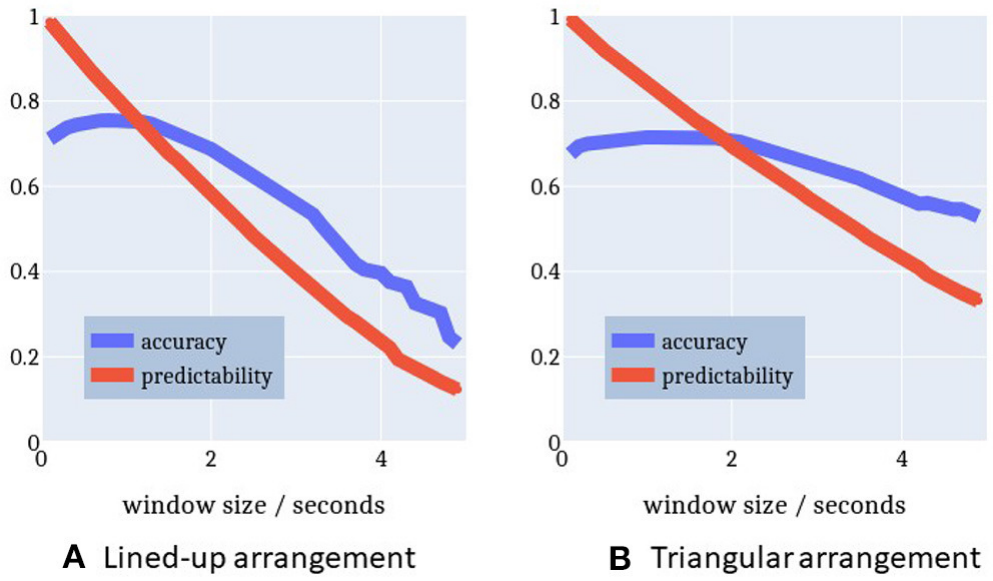
Relationship between time window (min = 0.1 s, max = 5 s) and performance measures. Time window and performance are inversely proportional to each other. A maximum accuracy for a reasonable predictability is reached at time window Δt = 0.9 s for the lined-up arrangement and Δt = 1.3 s for the triangle arrangement, respectively. **(A)** Lined-up arrangement. **(B)** Triangular arrangement.

Figure 12A highlights the relationship between the time window Δ*t*, accuracy, and predictability. With a longer time window both the prediction accuracy and the predictability decrease. A longer time window has the effect to include more observation samples belonging to a previous action rather than the current intention. This is sketched in Figure 9. As a result, either the log-probability threshold is not exceeded or an incorrect intention is recognized. There is a maximum accuracy at a time window of 0.9 s with a predictability of 77%. That is, after at least 23% of the action execution the right action is predicted in 78% of cases. Given this earliness, we can speak of intention recognition.

Figure 12B plots a similar relationship between time window and performance for the triangle-shaped arrangement. The optimal time window size here is 1.3 s with an accuracy of 75% (chance level = 16.7%) and a predictability of 78%. The accuracy curve seems to be flattened because the action execution times in this setup come with a larger spread. Especially, picking up the cylinders at positions 0 and 1 is more challenging and causes a longer execution time compared to the other sub-tasks in this triangle setup. This issue is apparent also in Figure 11B with more distant whiskers and extended boxes for *pick_0* and *pick_1*.

Furthermore, the plots in Figures 11, 12 confirm the observations described in section 3.4. The gaze behavior seems to be independent of the spatial arrangement of the objects in the scene. This fact is very well-represented by the HMMs, which have been trained only on lined-up arrangement data, but perform almost as well on the triangle arrangement data.

**Figure 13:**
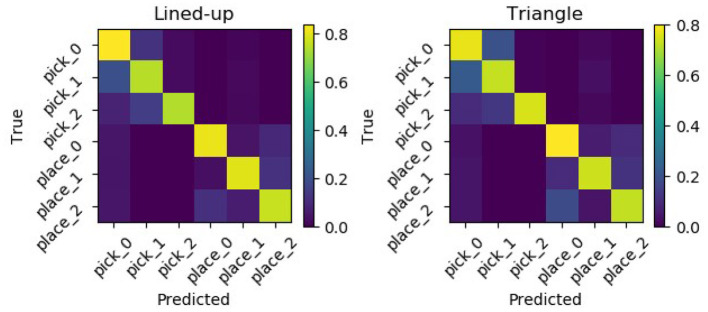
Normalized confusion matrices for the two picking arrangements. Errors are mostly made mistaking neighboring locations but still classifying the task correctly.

Moreover, Figure 13 shows the confusion matrices for the two tested spatial arrangements. It can be appreciated that when the model delivers a wrong prediction it usually mistakes neighboring picking or placing locations, but still correctly identifies the task.

### 4.3. An Alternative Model: The Semantic GHMM

Our hypothesis in designing the behavioral experiment and the model presented above was that the object positions and configuration would have an effect on the observed sensorimotor behavior. Yet, both the results of the behavioral analysis (cfr. Figures 5, 6) and the results of the modeling of separate action-object intentions show how gaze patterns are pretty similar across picking positions and configurations and how the models even generalize well to a new configuration with different picking positions. This suggests that rather the current motor primitive (pick or place), represented at a symbolic, semantic level, determines a prototypical sensorimotor pattern, which gets further specified by the motor system depending on the current situation (motor intentions as put forward by Pacherie, 2008). Yet, these further adjustments are at an intra-class level, preserving the general inter-primitive discriminability. This is probably specifically the case in the simplified context we have worked with here, where no real grasping is executed but still, the gripper needs to be placed correctly on the cylinder to allow a firm grasp or to achieve a stable placing down. The model proposed above has further the limitation of scalability: if more pick and place targets were added to the scene, possibly even dynamically appear or disappear, new models would need to be instantiated and trained, while also the feature vector to the models being correspondingly adapted every time. The same would of course occur if a further action would be added to the mix, with every possible combination of object and action being explicitly modeled and trained.

For these reasons, we also devised an alternative, semantic model to be tested against the first model. In this case, just two models are instantiated and trained, one for the pick and one for the place action. The same observations as used in the previous approach have been translated into a flexible object- and action-based arrangement of the feature vector fed to each GHMM model. Thus, the models receive always the same number of features, regardless of the number of objects in the scene. Assuming that *n* objects and *m* placing options are present in the scene and that the corresponding AoIs come labeled as either “pickable” or “placeable” candidates, to get the likelihood that object *i* (or at position *i*) is currently the pick target, the following vector is fed the *pick* GHMM:

(2) $$\begin{array}{l} F_{t}=\{\text{P}(\text{AoI}=a_{i}|g_{t}),\sum_{j\neq i}\text{P}(\text{AoI}=a_{j}|g_{t}),\sum_{k=1}^{m}\text{P}(\text{AoI}\\ \;=b_{k}|g_{t}),\text{P}(\text{AoI}=Robot|g_{t}),grasping\_state_{t})\}\;. \end{array}$$

and to get instead the likelihood that coaster *i* (or position *i*) is currently the place target, the following vector is fed the *place* GHMM:

(3) $$\begin{array}{l} F_{t}=\{\text{P}(\text{AoI}=b_{i}|g_{t}),\sum_{j\neq i}\text{P}(\text{AoI}=b_{j}|g_{t}),\sum_{k=1}^{n}\text{P}(\text{AoI}\\ \;=a_{k}|g_{t}),\text{P}(\text{AoI}=Robot|g_{t}),grasping\_state_{t})\}\;. \end{array}$$

**Figure 14:**
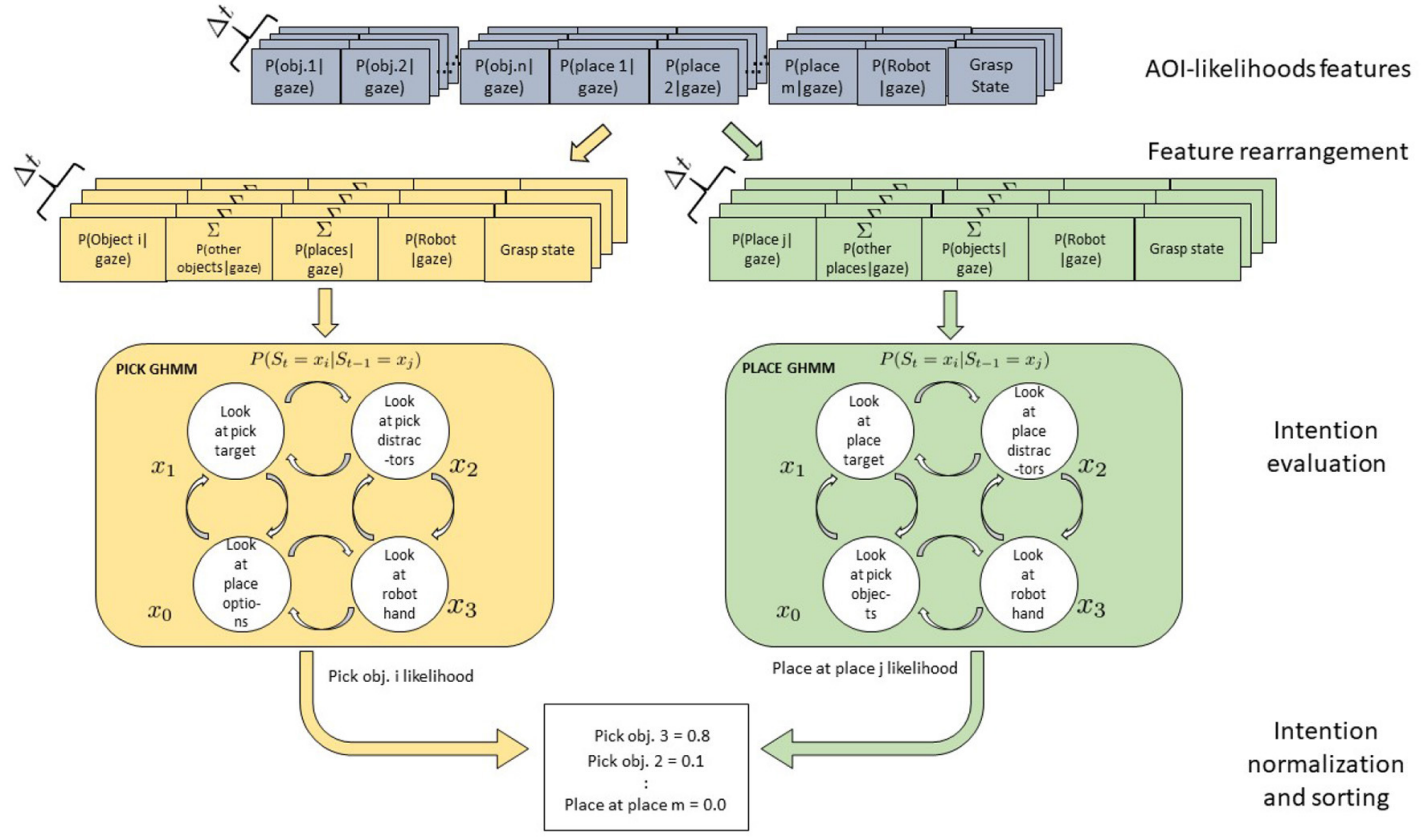
Gaussian Hidden Markov Models for a Pick-and-Place-Task. Four hidden states *x* are shown which might represent the perceptual state of the user looking at the object to be picked, at the robot, at the placing position target, or at the teleoperated robotic hand. Arrows between states represent transition probabilities: for the sake of legibility here only transitions between adjacent states are shown but actually all states are fully connected. Two models are defined for the pick and place intentions. Each model receives as input a vector of rearranged AoI likelihoods, with the first element representing the object to be tested for pick (place), while the second sums up the AoI likelihood of the other pick (place) distractors. The third element sums the features of the other objects relevant for the other action, while the robot and the grasping state features stay the same.

The two models were instantiated with four hidden states each (representing *looking at a pick* or *place target*, wandering with the gaze on any *distractor for pick* or *for place* or *looking at the robot hand*) and trained and tested with the same data as the first model. Any time a new sample is available from the AoI analysis, the two models are submitted *n* and *m* differently arranged feature vectors, respectively, and produce as many likelihood scores, with the highest-ranking taken as the estimated intention. This process is exemplified in Figure 14.

**Figure 15:**
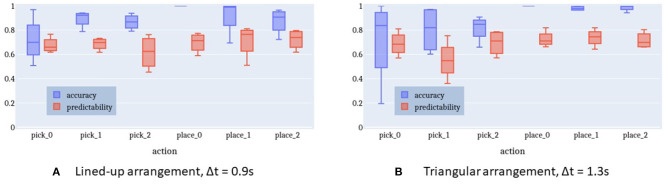
Accuracy and predictability of online intention recognition for lined-up arrangement and triangle shape arrangement in the semantic model. **(A)** Lined-up arrangement, Δt = 0.9 s. **(B)** Triangular arrangement, Δt = 1.3 s.

**Figure 16:**
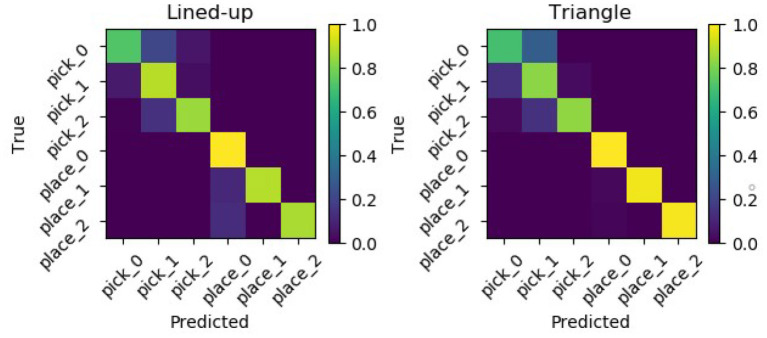
Normalized confusion matrices from the semantic GHMM for the two used spatial arrangements.

Results show that with the semantic models, the accuracy increases reaching a mean value of 88.0% and of 89.7% for the lined-up and triangular configuration, respectively (see Figure 15). On the one hand, the lower number of states (4) used in the semantic model might have contributed to the increase of the recognition accuracy. Indeed, although the naïve model uses one more state than the semantic model and this might fit the training data better, a higher number of states can unnecessarily overcomplicate the model and produce overfitting. On the other hand, the two semantic models have access to more training data w.r.t the six naive models: in general they can abstract better as to what defines a pick or a place action across the different targets. Considering the normalized confusion matrices depicted in Figure 16, in this case, no mistake is made between the two actions: the semantic models seem to be able to better learn the importance of the grasping state feature in discriminating the two actions, as further shown in the next subsection.

### 4.4. Effect of Grasping State on Performance

**Figure 17:**
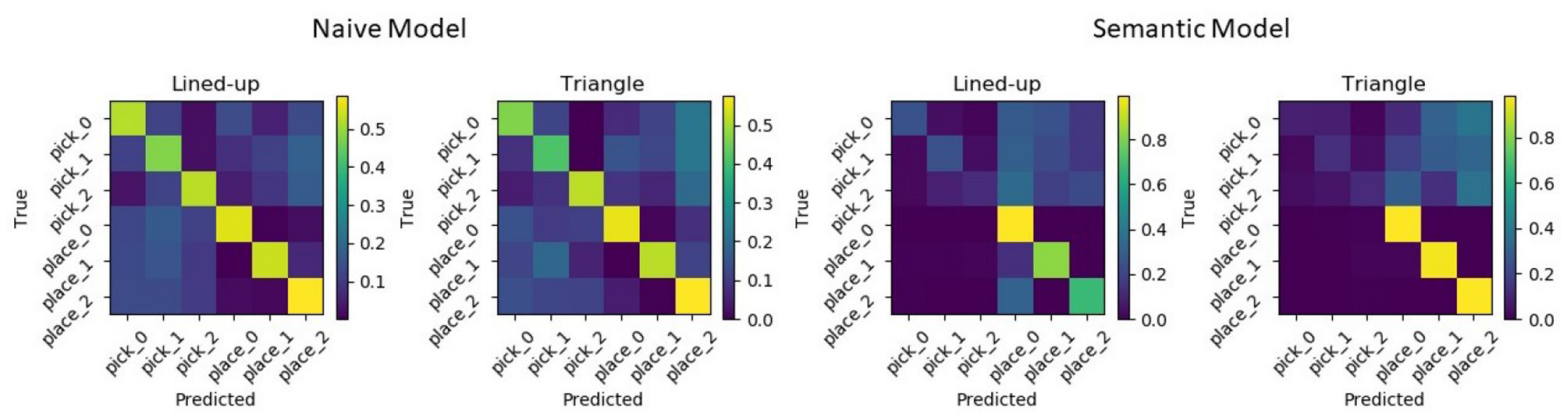
Normalized confusion matrices for the naive (left) and semantic (right) models for both configurations when training and testing the model without the grasping state feature. The semantic model achieves a slightly higher overall accuracy but fundamentally recognizes highly accurately just the placing intentions.

Assessing the model performance against a 16.7% chance level could be misleading since the grasping state constitutes a powerful binary cue to tell the two actions apart and hence a 33.3% chance level would be a fairer assessment. For this reason, both models were trained and tested again without the grasping state feature. Results with and without this feature are reported in Tables 1, 2. The accuracy substantially decreases for both models, but still remains on an above-chance level. The predictability rises almost at ceiling levels, probably because the models consider at any time the current fixation as indicative enough of the current intention, irrespective of its compatibility with the current grasp state, and outputs the corresponding intention. While on an overall level the naive and the semantic models achieve a similar accuracy of above 50%, it can be seen in the confusion matrices in Figure 17 that the naive model somehow manages to differentiate between the two different actions while the semantic model basically classifies most intentions as place intentions. A possible explanation could be that without the grasping cue or any other common-sense prior knowledge about picking and placing, the semantic models can only generally infer an intention to interact with an object. In this case, the “place” model, which relies on clearer, longer fixations on one target (see Figure 6), is the most confident about its predictions, while the “pick” model sees the gaze likelihood distributed among more objects. The naive models, on the other hand, which were separately trained on the single object/locations, manage to retrieve some of the regular patterns of each single intention. Still, it is reasonable to expect that with a larger dataset, both models could better learn the scanpath differences evident in Figure 6 and better discriminate between the two actions just by means of gaze features.

**Table 1:** Accuracy for the naive and semantic GHMM models with and without the grasping state feature.

**Naive model**	**With GS (%)**	**Without GS (%)**	**Semantic model**	**With GS (%)**	**Without GS (%)**
Lined-up	78.3	53.5	Lined-up	88.0	54.6
Triangular	75.3	51.5	Triangular	89.7	58.8

**Table 2:** Predictability for the naive and semantic GHMM models with and without the grasping state feature.

**Naive model**	**With GS (%)**	**Without GS (%)**	**Semantic model**	**With GS (%)**	**Without GS (%)**
Lined-up	77.1	98.5	Lined-up	70.5	98.5
Triangular	78.2	99.0	Triangular	71.9	99.2

### 4.5. Comparison to Active Fixation-Based Approaches

The specific nature of our setup makes it difficult to compare our system to other approaches presented in the literature, since often either natural eye-hand coordination (Haji Fathaliyan et al., 2018; Wang et al., 2020) or no eye-hand coordination at all (Huang and Mutlu, 2016) is used for intention recognition (see section 2). In teleoperation, especially in the context of assistive technologies, the user is often required to actively fixate the object of interest for a certain amount of time in order to trigger an associated action (Wang et al., 2018; Cio et al., 2019; Shafti et al., 2019). We compare here our system to such approaches, to verify the advantage of a probabilistic framework over a deterministic, sensory-driven one. To this end, we computed the classification performance when considering a fixation as a time window Δ*t* where the same AoI had consistently the highest likelihood and took as prediction the corresponding intention (e.g., if *a*_0_ was fixated for Δ*t* s the prediction would be “pick at *a*_0_”). Note that this method assumes that each object is associated with only one action (either pick or place), thus it would not mistake the two actions. We used Δ*t* of different sizes to compare the fixation performance to our model (Δ*t* = 0.9 for the lined-up, Δ*t* = 1.3 for the triangular configuration), to the approach by Shafti et al. (2019) (Δ*t* = 1.5 s), and to the approach by Wang et al. (2018) (Δ*t* = 2 s). It must be stressed that in our case the users were not actively fixating the objects to make their intentions legible: when fixating naturally, it is rarely the case that fixations this long occur, hence we further tested a shorter window Δ*t* = 0.5. Accuracy and predictability results are reported in Table 3. Even if this system reaches an accuracy at times comparable with that of our model without grasping state feature, still the predictability is considerably lower. That is, only when a fixation on an object is ongoing for sufficient time a prediction is available, while most of the time no intention is predicted. Indeed, considering a shorter Δ*t* = 0.5 produces the best accuracy and predictability scores. In contrast, our model emits a more reliable prediction earlier in time and maintains it even when the gaze is not on the target object since transitions are accounted for.

**Table 3:** Accuracy and predictability of the AoI features considering different fixation times for comparison with the fixation times of 1.5 s used by Shafti et al. (2019) and of 2 s used by Wang et al. (2018).

Configuration (fixation time)	Lined-up (Δ*t* = 0.5)	Triangle (Δ*t* = 0.5)	Lined-up (Δ*t* = 1.5)	Triangle (Δ*t* = 1.5)	Lined-up (Δ*t* = 2.0)	Triangle (Δ*t* = 2.0)	Lined-up (Δ*t* = 0.9)	Triangle (Δ*t* = 1.3)
Accuracy	66.0%	62.4%	52.1%	54.3%	42.6%	48.8%	61.0%	56.3%
Predictability	47.0%	53.8%	24.1%	35.0%	16.1%	28.4%	37.0%	37.9%

*Since in general shorter fixation times occurred in the trials, also a shorter 0.5 s interval was tested. A further comparison is done using a fixation time equal to the window times used in our model (Δt = {0.9, 1.3s})*.

## 5. Discussion and Conclusions

We presented a study aimed at investigating eye-hand coordination and gaze-based intention recognition during teleoperated pick-and-place tasks. The ultimate goal is to transfer such intention recognition into a shared autonomy architecture. To this end, in this first study on the one hand data was collected and analyzed in order to have a baseline characterization of user behavior in a fully teleoperated modality. On the other hand, collected data was used to train a model flexible enough to work with different users and in possibly different settings.

In teleoperation contexts natural eye-hand coordination is somehow disrupted since action is mediated by an input controller and executed by a robotic system. This arrangement upends the internal forward and inverse model predictions and places a further monitoring load on the visual system. Hence, as first studies besides this have shown (Aronson and Admoni, 2018; Aronson et al., 2018), investigating eye-hand coordination during teleoperation can shed light on the user's specific sensorimotor behavior and needs in such setting, prompting better design and models for intention recognition in such systems. Still, in contrast to those studies aimed at assistive applications, we strove for a more natural control input based on motion tracking. In this way, we aim to elicit and exploit patterns of eye-hand coordination similar to those used in real grasping and acting. The analysis of eye and hand behavior has revealed that, although participants in most cases managed to successfully operate the gripper in the pick-and-place task, still some positions required more grasp attempts and longer reaching times. This is in part due to the impaired depth estimation on the screen, however, the difficulty in aligning the gripper with the cylinder in the furthest position or in avoiding bumping into cylinder 1 to grasp in position 2 in the triangular arrangement required extra care and slowed down the movement. Moreover, while the gaze behavior showed some similarities with natural eye-hand coordination, e.g., locating and guiding the hand to the target of the next proximal intention (Land et al., 1999), we found that both in the reaching and in the transport phase the robot gripper was looked at for quite some time, differently from what happens when grasping with the own hand (Johansson et al., 2001). This represents an indicator that the participant preferred to visually monitor the gripper movement in the absence of the usual proprioceptive coordination and tactile feedback. Furthermore, the object held in hand was looked at also after the grasping was triggered, again something that does not happen in natural eye-hand coordination, since tactile feedback confirms the expected contact event and successful grasping (Johansson and Flanagan, 2009). In this teleoperation scenario instead, the grasp had to be confirmed visually, hence the gaze lingered on the picked object and only after seeing the object moving along with the hand, moved on to the next distal intention (i.e., the placing position). Still, this kind of measures offers an insight into the user experience of the teleoperation task: as long as the uncertainty about the task execution is high, the gaze is less anticipative and lingers there where further information needs to be acquired to carry out the task. Although some of these issues could be mitigated with longer training, allowing the user to master the new visuomotor mapping and task (Sailer et al., 2005), an intention recognition model embedded in a shared autonomy architecture that could adjust the robot movement and grasping pose to reliably produce the intended grasp would shorten these training times. This would allow a more natural eye-hand coordination and relieve the gaze system of monitoring every sub-task unfolding and transition with extra care. That is, an effective shared autonomy system would be validated by shorter execution times, fewer failed grasp attempts, and more anticipative gaze behavior with less time spent monitoring the grasped object and the robot gripper. This would confirm that the user trusts the robotic partner to correctly infer and assist with the current intention but that their sense of agency is preserved since they anticipate the next subtask in their plan (see on this the discussion in Haji Fathaliyan et al., 2018).

Apart from these considerations, as shown for a different task (Keshava et al., 2020), we also found that the gaze behavior still was reliably different across tasks and could be hence learned and predicted effectively. To this end, a Hidden Markov Model was first devised for each of the intentions to be recognized. The normalized likelihoods of the gaze (represented as a Gaussian distribution) to be on each of the objects in the scene along with the grasping state were considered as emissions of the HMM. The system was trained on pick-and-place tasks from two users and then tested on similar unseen sequences from the two users plus two other users. Considering a time window of 0.9 s where emissions are accumulated and then scored by the six GHMMs, the system achieves a well above chance accuracy across all tasks, returning a prediction as early as after seeing 22% of the current action, on average. Here, the concept of predictability, indicating the portion of the task for which an estimate is available, relates to that of observational latency. As pointed out in Wang et al. (2020), even a very accurate prediction is of very low utility if it is not delivered in time for the system to plan and execute a supporting operation before the user has carried out the action themselves. The generalizability of the system was further tested on a different geometrical configuration of the pick task, delivering comparable accuracy and predictability. Even more accurate results were obtained by a second intention recognition scheme, which modeled the two basic actions (pick and place) and scored the likelihood of each picking or placing target by appropriately arranging the features representing the likelihood of the gaze on the different AoIs. Also, in this case, generalization was higher with new users and configurations, while practically no confusion between the two classes was observed. This kind of model offers also the possibility of scaling up the system to new picking objects and support surfaces, not previously seen during training: the dimension of the feature vector fed to the GHMMs stays in fact constant and the arrangement of the features determines which object is evaluated as pick/place target. The amount of gaze distribution captured by other objects of the same category (which should still be comparatively low compared to the real target) and by all those of the other category is indeed considered as two collective features, independent of the number of present objects or support surfaces.

A test without the binary grasping state feature, yet, showed a less consistent performance of the semantic model with respect to the naive model: the semantic model perfectly recognized the place intentions but mostly misclassified the pick intentions as place intentions. This might be due to the fact that the semantic model relies more strongly on the grasping state to determine the action and uses the gaze data to infer the object of interest, while the six naive GHMMs better learned the specificity of the scanpaths in the different conditions.

In any case, considering the similar semantic distributions of gaze time within equivalent sub-tasks and across spatial configurations, these results suggest that there is a certain invariance in the gaze patterns. These are mainly shaped by the general sub-task at a higher level. At least in simple manipulation tasks and object configurations, sequences of gaze glances at objects are more heavily determined and constrained by the current subtask structure (pick vs. place), once the target is specified, rather than by the contingent spatial setup. That is, also the oculomotor plan subserving and directing the motor plan seems to reflect the syntactic structure of action (Pastra and Aloimonos, 2012).

These are promising results for the further development of our intention recognition system and its embedding in a real-world shared autonomy scenario. Current and future work is going to expand both the training and testing sets with multiple participants as well as considering more and different objects and tasks. A richer dataset with data from naive participants would indeed provide a better characterization not only of the users' sensorimotor behavior in itself, but it would indeed allow testing for learning effects within each participant and individual differences between participants. This would also help to investigate the co-adaptation process between human and robotic systems (Gallina et al., 2015). On the one hand, indeed, visuomotor adaptation to the new environment produces effective motor learning enabling the user to better handle the initially unfamiliar sensorimotor mapping. This effect could override some of the features learned by the intention recognition framework and should hence be accounted for. On the other hand, it should be investigated how different users cope on a sensorimotor level with the same task. This could both help to understand the generalization limits across users of a pre-trained model and to identify possibilities for user customization. Experimenting with a more complex scenario in terms of number and configuration of objects and support surfaces would very likely affect the high accuracy observed in this study, yet would also offer insight into meaningful ways to effectively assist the user and on ways to tackle the trade-off between accuracy and observational latency also downstream, at the behavior control level.

## Data Availability Statement

The dataset presented in this article is not readily available: considering the small number of participants, the dataset was not intended for public dissemination. Requests to access the dataset can be directed to Anna Belardinelli (anna.belardinelli@honda-ri.de).

## Ethics Statement

The studies involving human participants were reviewed and approved by Honda Bioethics Committee. Written informed consent for participation was not required for this study in accordance with the national legislation and the institutional requirements.

## Author Contributions

SF and AB designed and conducted the study. SF devised the computational models. SF and AB analyzed the results. AB performed the behavioral data analysis and interpretation and wrote the main manuscript.

## Conflict of Interest

When conducting the study both authors were employed by the Honda Research Institute Europe GmbH. During the writing of the manuscript, SF moved to Siemens AG.

## Footnotes

^1^To avoid confusion with terms sometimes used interchangeably, sometimes meaning different things, we here refer to task as the overarching ongoing activity, e.g., pick and place, while intention implies the commitment to perform the current proximal action/sub-task, e.g., reaching to grasp.

^2^http://gazebosim.org/

^3^The gaze was mapped in this way since in a later stage we plan to move the simulation into a virtual reality headset with embedded eye tracking and the gaze mapping on the scene can stay unaltered.

^4^https://hmmlearn.readthedocs.io
